# Estrogen-Dependent Regulation of FDPS in the Mouse Uterus and Its Expression in Endometrial Cancer

**DOI:** 10.3390/ijms27031559

**Published:** 2026-02-05

**Authors:** Yeonju Suh, Byeongseok Kim, Joohee Kim, Jimin Lee, Sangok Park, Soohyung Lee, Man Ryul Lee, Hoi Chang Lee, Youngsok Choi

**Affiliations:** 1 Department of Stem Cell and Regenerative Biotechnology, Konkuk University, Seoul 05029, Republic of Korea; syj93129@naver.com (Y.S.); qufsksrlaqud@naver.com (B.K.); ysjh06029@naver.com (J.K.); 3136jm@naver.com (J.L.); sangok731@naver.com (S.P.); leseichi@naver.com (S.L.); 2 Major in Stem Cell and Regenerative Biotechnology, School of Advanced Biotechnology, Konkuk University, Seoul 05029, Republic of Korea; stemopia@konkuk.ac.kr; 3 The Institute of Advanced Regenerative Sciences, Konkuk University, Seoul 05029, Republic of Korea; 4 Department of Obstetrics and Gynecology, Feinberg School of Medicine, Northwestern University, Chicago, IL 60611, USA; hoi.lee@northwestern.edu

**Keywords:** farnesyl diphosphate synthase, uterus, endometrium, estrogen, endometrial cancer

## Abstract

The uterus is a dynamic organ in which the endometrium undergoes cyclic processes of proliferation, shedding, and regeneration under the influence of estrogen and progesterone. In particular, estrogen regulates the proliferation and differentiation of the endometrium and plays an important role in the development of gynecological diseases such as endometrial cancer. Farnesyl diphosphate synthase (FDPS) is a key enzyme involved in the mevalonate pathway, catalyzing the synthesis of farnesyl pyrophosphate (FPP), which plays an essential role in cholesterol biosynthesis and protein prenylation. In this study, we demonstrated using an in vivo mouse model that the expression of FDPS is regulated by estrogen. FDPS expression was specifically elevated during the proestrus stage of the estrous cycle and subsequently decreased. In ovariectomized (OVX) mice, FDPS expression was significantly increased 24 h after estrogen treatment, whereas this response was suppressed by treatment with the estrogen receptor alpha (ERα) antagonist, ICI 182,780. Although FDPS expression has been reported in various cancers, its role in endometrial cancer remains unclear. Histological and cellular analyses revealed that FDPS is highly expressed in human endometrial cancer tissues and in the endometrial cancer cell line Ishikawa, where it contributes to cell proliferation. These findings suggest that FDPS may play a role in the survival and growth of endometrial cancer cells. This study provides new insights into the potential function of FDPS in the uterus and suggests that targeting FDPS may represent a promising therapeutic strategy for endometrial cancer.

## 1. Introduction

The uterus is a central organ in female reproduction, consisting of three layers: the endometrium, myometrium, and perimetrium. Among these, the innermost endometrium undergoes dynamic regulation by estrogen, leading to cyclical changes in proliferation, shedding and regeneration throughout the estrous cycle. Mice experience a four-stage estrous cycle—proestrus, estrus, metestrus, and diestrus—which is tightly regulated by estrogen and progesterone. Estrogen levels peak during the proestrus phase, gradually decline thereafter, and rise again at the end of diestrus [1].

Estrogen plays a central role in regulating the development and physiological functions of the female reproductive system. It is mainly synthesized in the ovaries and mediates its actions via estrogen receptors (ERs). ERs exist in two forms, ERα and ERβ, with ERα being predominantly expressed in the uterus [2]. ERα serves as the principal receptor regulating endometrial proliferation and differentiation, and estrogen-induced uterine responses are mainly mediated through ERα [3]. Moreover, estrogen is deeply involved in the pathogenesis of several gynecological diseases, such as breast cancer, endometrial cancer, and endometriosis [4].

Farnesyl diphosphate synthase (FDPS) is a key enzyme in the mevalonate pathway that catalyzes two-step condensation, leading to farnesyl pyrophosphate (FPP) formation. Isopentenyl pyrophosphate (IPP) and dimethylallyl pyrophosphate (DMAPP) condense to form geranyl pyrophosphate (GPP), which then undergoes an additional condensation with IPP to produce FPP. FPP serves as a substrate for cholesterol biosynthesis via squalene synthase, thereby participating in the biosynthesis of steroid hormones such as estrogen and progesterone. It also serves as a precursor for geranylgeranyl pyrophosphate (GGPP), which is essential for protein prenylation [5]. Dysregulated FDPS expression has been reported to contribute to the development of multiple types of cancers [6].

Endometrial cancer (EC) represents one of the most prevalent gynecologic malignancies globally, and its incidence has been steadily increasing [7]. EC is classified into estrogen-dependent type 1 and estrogen-independent type 2 based on molecular and histopathological characteristics. Type 1 EC primarily consists of well-differentiated (grade 1–2) endometrioid adenocarcinomas, whereas type 2 EC includes grade 3 endometrioid adenocarcinomas, serous, clear-cell, undifferentiated carcinomas, and carcinosarcomas [8]. Endometrioid adenocarcinoma is the most common histological subtype, accounting for approximately 75–80% of all EC cases, with more than 85% of these being low-grade (grade 1–2) tumors [9].

Although FDPS expression has been investigated in various tissues and cancers, its expression pattern and regulatory mechanisms in the uterus and endometrial cancer remain largely unexplored. Considering that estrogen plays a pivotal role in regulating endometrial proliferation and differentiation, elucidating how FDPS expression is influenced by estrogen may provide important insights into both physiological and pathological regulation of uterine function. Therefore, this study aimed to identify estrogen-responsive FDPS expression in the mouse uterus using an in vivo model and to examine the effects of FDPS expression on cellular behavior in the human endometrial cancer cell line Ishikawa, thereby providing a fundamental basis for developing new therapeutic strategies for uterine disorders.

## 2. Results

### 2.1. Transcriptomic Profiling of Estrogen Response in the Mouse Uterus

To identify estrogen-responsive genes in the mouse uterus, we performed a transcriptomic analysis as an initial exploratory step. Heatmap analysis was performed to identify genes that showed significant expression differences among the various groups. The significance threshold was set at |logFC| > 1 and FDR < 0.05.

**Figure 1:**
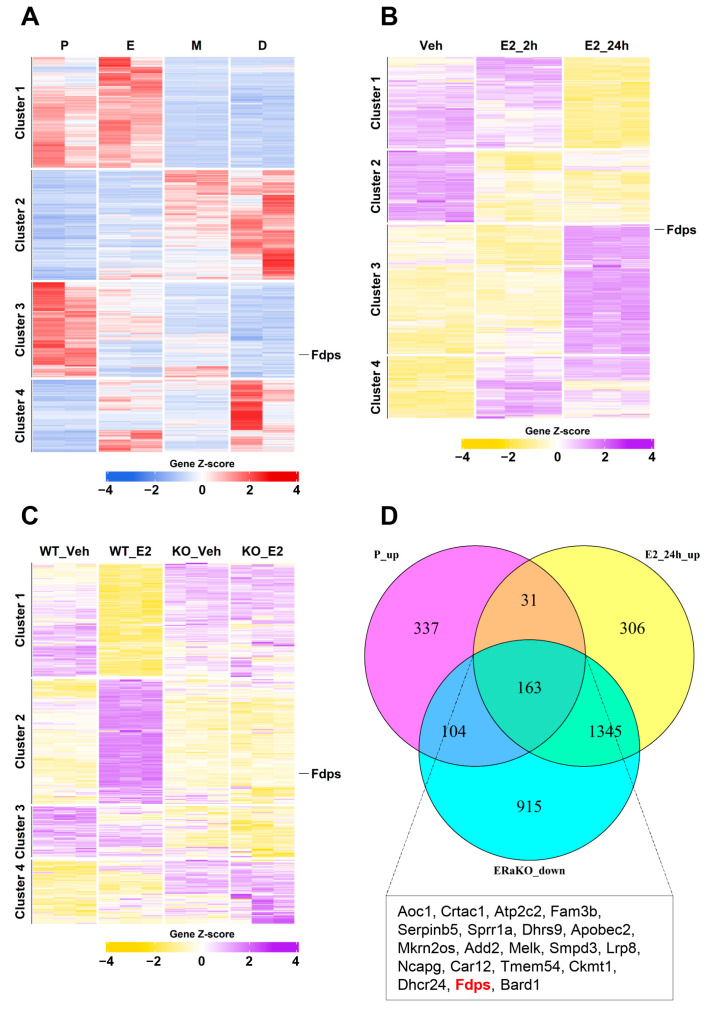
Transcriptomic analysis of estrogen-responsive genes in the mouse uterus. (**A**) Heatmap of bulk RNA-seq data (GSE241420) indicating differentially expressed genes during estrous cycle. P, proestrus; E, estrus; M, metestrus; D, diestrus. (**B**) Heatmap of microarray data (GSE53812) showing gene expression in the uterus of ovariectomized (OVX) mice treated with E_2_. Veh, vehicle (E_2_ treatment for 0 h); E2_2h, 2 h; E2_24h, 24 h. (**C**) Heatmap of microarray data (GSE23072) indicating differentially expressed genes in the uteri of OVX mice with ERα knockout (ERαKO) after E_2_ treatment for 0 h and 24 h. (**D**) Venn diagram illustrating the overlap of genes that are upregulated in the proestrus phase and after 24 h of E_2_ treatment, but remain unchanged in the ERαKO group. The box below the Venn diagram displays a representative list of the top 20 genes identified from the intersection, with *Fdps* highlighted in red.

To first identify genes that are naturally regulated by endogenous estrogen, a heatmap analysis was conducted using uterine transcriptomes from the four stages of the mouse estrous cycle: proestrus (P), estrus (E), metestrus (M), and diestrus (D). A total of 2585 significantly altered genes were identified, among which 635 genes in cluster 3, characterized by increased expression during the proestrus stage with the highest estrogen level, were selected for further analysis (Figure 1A).

Using the same analytical approach, to examine the response to exogenous estrogen, we next examined the gene expression patterns in ovariectomized (OVX) mice from the vehicle (Veh), estrogen 2-h (E2_2h), and estrogen 24-h (E2_24h) treatment groups. This analysis identified 5050 significant genes, and particular attention was given to the 1845 genes in cluster 3, which showed high expression levels at 24 h after E_2_ treatment (Figure 1B).

To further identify estrogen-induced genes that are mediated through estrogen receptor alpha (ERα), heatmap analysis was performed using uterine samples from ERα knockout (ERαKO) mice. A total of 7155 significant genes were identified, among which 2527 genes in cluster 2 exhibited significant induction at 24 h after E_2_ treatment in wild-type (WT) mice but showed no expression changes in ERαKO mice (Figure 1C).

Finally, by integrating these three datasets to isolate core ERα-responsive genes, we identified a subset of 163 genes that were highly expressed in both the proestrus and E2_24h groups but showed no induction in ERαKO uteri. These genes were visualized using a Venn diagram (Figure 1D). Among them, *Fdps* ranked 19th in logFC when comparing Veh vs. E2_24h, placing it within the top 12% of genes with the strongest effect size.

### 2.2. FDPS Expression in Mouse Uterus Throughout the Estrous Cycle

Based on the transcriptomic profiling results in Figure 1, we hypothesized that FDPS is a key estrogen-responsive gene whose expression is dynamically regulated by estrogen signaling in the mouse uterus. To test this hypothesis, we conducted a series of in vivo experiments to examine whether FDPS expression correlates with estrogen levels.

**Figure 2:**
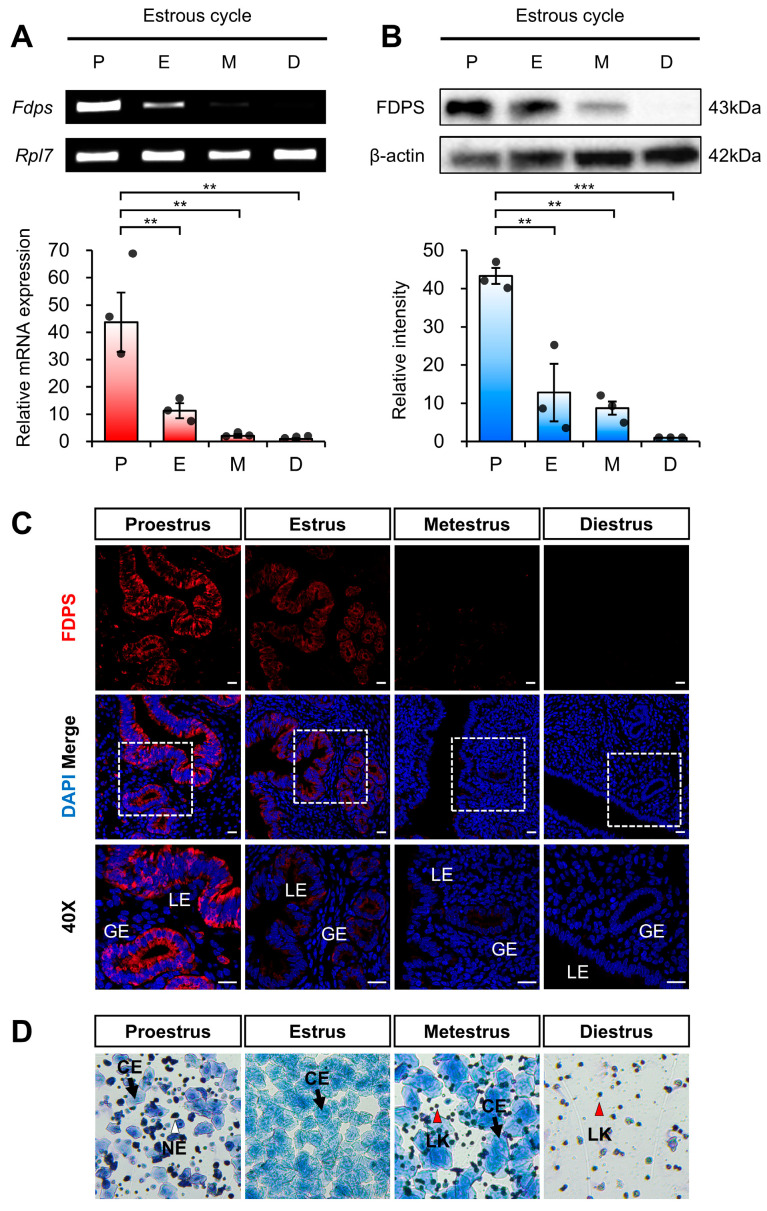
Expression pattern of FDPS in the mouse uterus across the estrous cycle. (**A**) RT-PCR and qPCR analyses of *Fdps* in mouse uteri at each stage of the estrous cycle. *Rpl7* was used as an internal control. P, proestrus; E, estrus; M, metestrus; D, diestrus. (**B**) Western blot analysis of FDPS protein expression in mouse uteri at each stage of the estrous cycle. β-actin was used as an internal control. (**C**) Immunofluorescence staining of the mouse uteri at each stage of the estrous cycle using FDPS antibody. Relative fluorescence intensity of FDPS was quantified (Supplementary Figure S1A). White boxes indicate the magnified regions. Scale bar = 20 μm. LE, luminal epithelial cells; GE, glandular epithelial cells. (**D**) Vaginal smear assay to determine the estrous cycle stages. Images were captured at 10× magnification. CE, cornified epithelial cell; NE, nucleated epithelial cell; LK, leukocyte. In all bar graphs, individual data points (*n* = 3) are shown as dots. Statistical significance: ** *p* < 0.01; *** *p* < 0.001.

To first assess estrogen-dependent regulation of FDPS under physiological conditions of endogenous estrogen, we analyzed its expression pattern across different stages of the mouse estrous cycle. Each stage of the estrous cycle was determined by vaginal smear analysis (Figure 2D). The expression of FDPS was assessed at both the mRNA and protein levels.

RT-PCR and qRT-PCR analyses revealed that *Fdps* expression was significantly increased during the proestrus stage, followed by a marked decrease as the cycle progressed through the estrus, metestrus, and diestrus stages (Figure 2A).

Consistent with the mRNA data, Western blot analysis also demonstrated a pronounced increase in FDPS protein expression during the proestrus stage (Figure 2B). In addition, immunofluorescence analysis showed that FDPS was predominantly localized to the luminal epithelial (LE) and glandular epithelial (GE) cells of the endometrium. The fluorescence intensity of FDPS was most prominent during the proestrus stage, gradually declining in the subsequent stages of the cycle (Figure 2C).

### 2.3. FDPS Expression in E_2_-Treated OVX Mouse Uterus

To determine whether FDPS is dynamically regulated in a time-dependent manner following estrogen stimulation, we examined its temporal expression pattern in ovariectomized (OVX) mice following exogenous estrogen administration. Ovariectomy was performed to eliminate endogenous hormones, including estrogen, thereby allowing the observed changes in FDPS expression to be specifically attributed to exogenous estrogen treatment.

**Figure 3:**
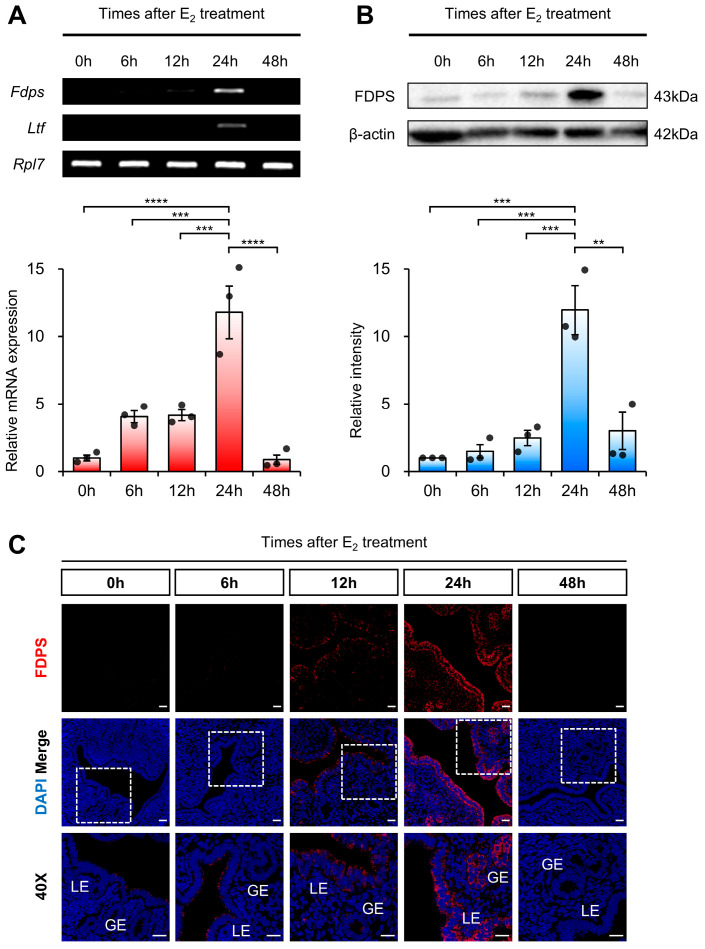
Expression of FDPS in the uterus of ovariectomized (OVX) mouse treated with E_2_. (**A**) RT-PCR and qPCR analyses of *Fdps* in OVX mouse uteri after E_2_ treatment. *Rpl7* was used as an internal control. (**B**) Western blot analysis of FDPS protein expression in OVX mouse uteri after E_2_ treatment. β-actin was used as an internal control. (**C**) Immunofluorescence staining of OVX mouse uteri after E_2_ treatment using an FDPS antibody. Relative fluorescence intensity of FDPS was quantified (Supplementary Figure S1B). White boxes indicate the magnified regions. Scale bar = 20 μm. LE, luminal epithelial cells; GE, glandular epithelial cells. In all bar graphs, individual data points (*n* = 3) are shown as dots. Statistical significance: ** *p* < 0.01; *** *p* < 0.001; **** *p* < 0.0001.

RT-PCR and qRT-PCR analyses revealed that *Fdps* expression was initially very low, gradually increased over time, and became significantly elevated at 24 h after E_2_ administration, followed by a subsequent decline. As a positive control, the expression of *Ltf (Lactotransferrin)*, a well-known estrogen-responsive target gene in the mouse uterus, was induced by E_2_ treatment, indicating the efficacy of the hormone stimulation (Figure 3A). *Fdps* expression showed no significant change 24 h after progesterone treatment compared with the vehicle group (Supplementary Figure S2).

A similar pattern was observed at the protein level. Western blot analysis showed that FDPS expression progressively increased after E_2_ treatment, peaking sharply at the 24-h time point, and then declined thereafter (Figure 3B). Consistent with the expression pattern observed during the normal estrous cycle, FDPS protein was predominantly localized in the LE and GE cells of the endometrium. At the 24-h peak, strong FDPS expression was detected in both cell types (Figure 3C).

### 2.4. Estrogen-Dependent Expression of FDPS Is Mediated by ERα

To determine whether the estrogen-induced expression of FDPS in the uterus is mediated by ERα, we examined FDPS expression following co-treatment with E_2_ and the ERα antagonist ICI 182,780.

**Figure 4:**
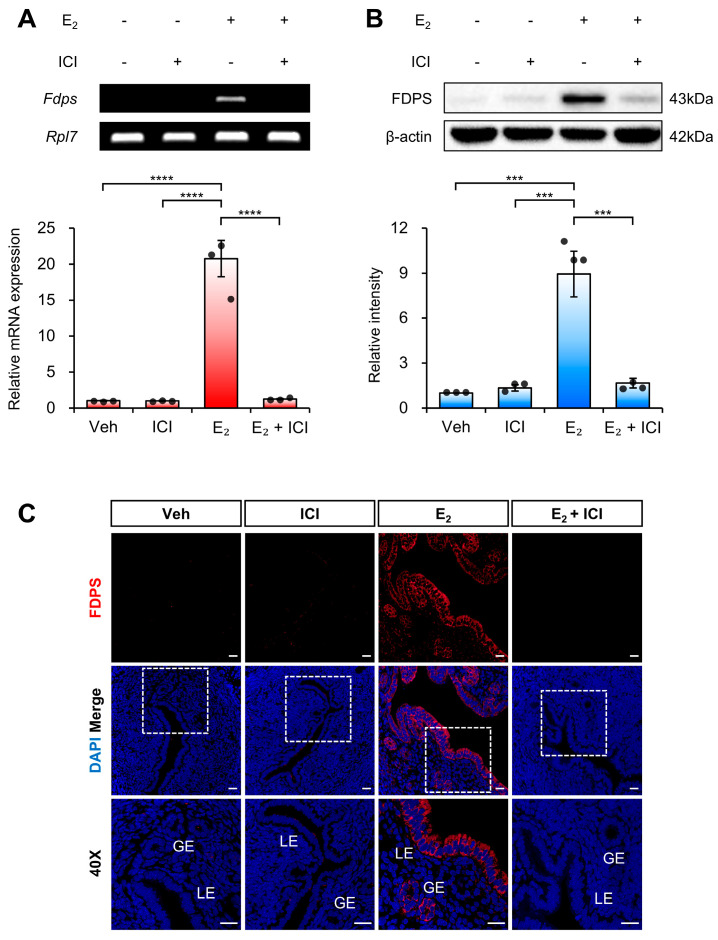
Expression of FDPS in the uterus of ovariectomized (OVX) mouse treated with ICI. (**A**) RT-PCR and qPCR analyses of *Fdps* in the uteri of OVX mice at 24 h after treatment with vehicle, ICI, E_2_ and E_2_ + ICI. *Rpl7* was used as an internal control. (**B**) Western blot analysis of FDPS protein expression in the uteri of OVX mice at 24 h after treatment with vehicle, ICI, E_2_ and E_2_ + ICI. β-actin was used as an internal control. (**C**) Immunofluorescence staining of the uteri of OVX mice at 24 h after treatment with vehicle, ICI, E_2_ and E_2_ + ICI using an FDPS antibody. Relative fluorescence intensity of FDPS was quantified (Supplementary Figure S1C). White boxes indicate the magnified regions. Scale bar = 20 μm. LE, luminal epithelial cells; GE, glandular epithelial cells. In all bar graphs, individual data points (*n* = 3) are shown as dots. Statistical significance: *** *p* < 0.001; **** *p* < 0.0001.

At the mRNA level, treatment with ICI alone significantly suppressed *Fdps* expression compared with the E_2_-only group. Moreover, co-treatment with ICI and E_2_ failed to induce the estrogen-dependent increase in *Fdps* expression observed with E_2_ treatment alone (Figure 4A).

A similar expression pattern was observed at the protein level (Figure 4B,C). These findings indicate that ERα plays a critical role in mediating the estrogen-dependent induction of FDPS expression in the uterus.

### 2.5. FDPS Expression in Endometrial Cancer

**Figure 5:**
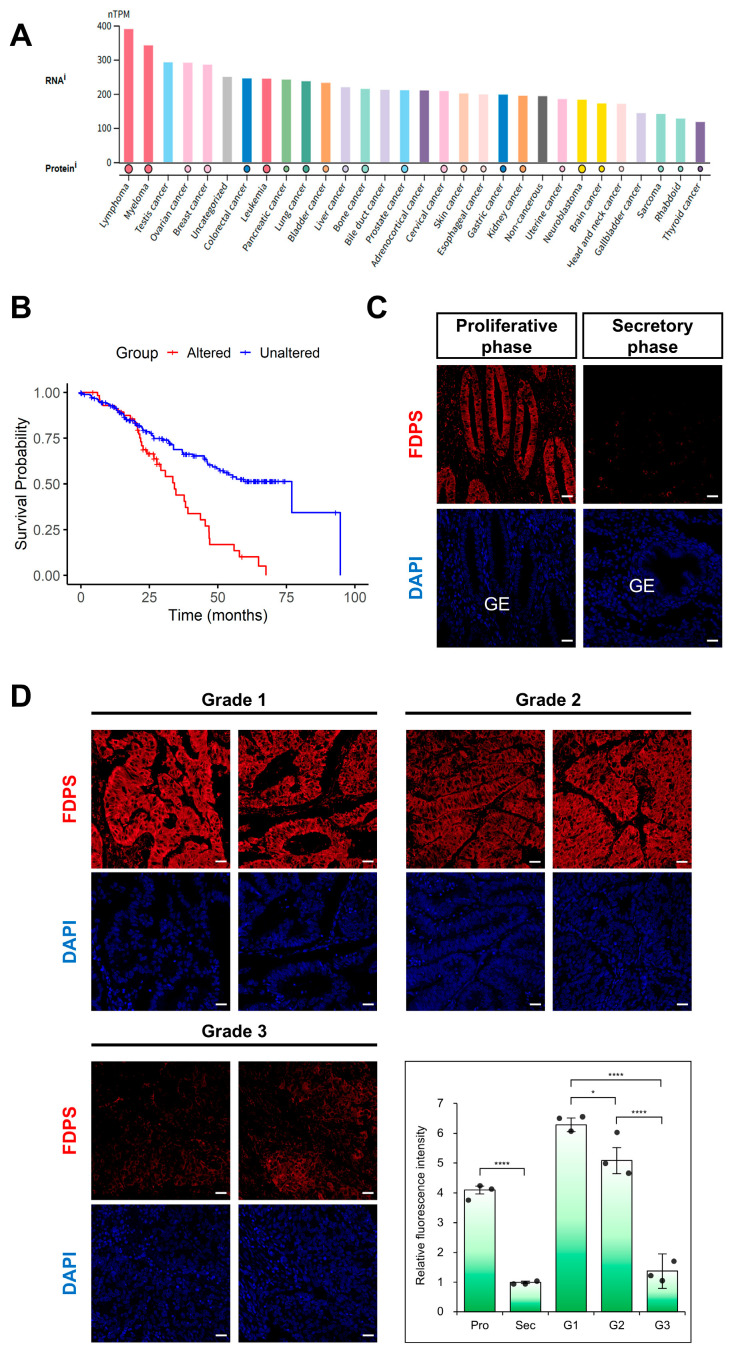
Expression of FDPS in human cancers and endometrial cancer tissues. (**A**) FDPS expression across human cancer cell lines. Expression data are shown as normalized transcript per million (nTPM). Data were obtained from The Human Protein Atlas (HPA). (**B**) Kaplan–Meier curves showing the overall survival of pan-cancer patients with FDPS alterations versus unaltered cases, obtained from cBioPortal (Pan-Cancer Analysis of Whole Genomes, ICGC/TCGA, Nature 2020). Log-rank *p* = 1.12 × 10^−5^. (**C**) Immunofluorescence staining of the normal human endometrium using an FDPS antibody. Scale bar = 20 μm. GE, glandular epithelial cells. (**D**) Immunofluorescence staining of human endometrioid adenocarcinoma tissues with different grades of differentiation using an FDPS antibody. Relative fluorescence intensity of FDPS was quantified. Scale bar = 20 μm. Pro, proliferative phase; Sec, secretory phase; G1, grade 1; G2, grade 2; G3, grade 3. In all bar graphs, individual data points (*n* = 3) are shown as dots. Statistical significance: * *p* < 0.05; **** *p* < 0.0001.

According to data from The Human Protein Atlas (HPA), FDPS showed high expression across various cancer types (Figure 5A). In addition, pan-cancer survival analysis using the cBioPortal database revealed that patients with FDPS gene alterations had significantly lower overall survival rate compared with those without alterations (log-rank *p* = 1.12 × 10^−5^) (Figure 5B).

Focusing on endometrial cancer (EC), immunofluorescence analysis was performed using tissue microarrays (TMAs). In the normal endometrium, FDPS was strongly expressed in glandular epithelial cells during the proliferative phase, whereas its expression was markedly reduced in the secretory phase (Figure 5C). This expression pattern was consistent with that observed in the mouse estrous cycle.

In endometrial cancer, FDPS expression levels differed depending on the degree of differentiation. High expression was observed in grade 1 (well-differentiated) tissues, while grade 2 (moderately differentiated) tissues showed intermediate expression levels between grade 1 and grade 3 (poorly differentiated) samples. In contrast, grade 3 tissues exhibited markedly decreased FDPS expression (Figure 5D).

### 2.6. Functional Characterization of FDPS Knockdown in Ishikawa Cells

**Figure 6:**
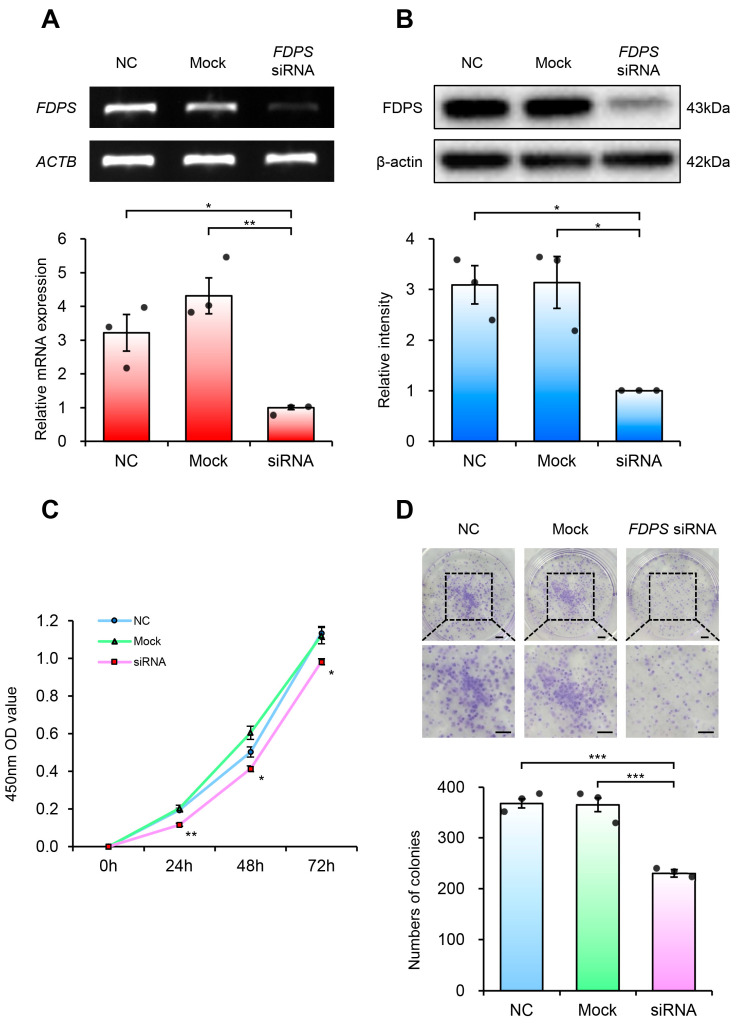
FDPS knockdown suppresses proliferation of endometrial cancer cells. (**A**) RT-PCR and qPCR analyses of FDPS in Ishikawa cells at 48 h after transfection with *FDPS* siRNA. *ACTB* was used as an internal control. (**B**) Western blot analysis of FDPS in Ishikawa cells at 48 h after transfection with *FDPS* siRNA. β-actin was used as an internal control. (**C**) CCK-8 assay showing the proliferation of cells transfected with *FDPS* siRNA at 0, 24, 48, and 72 h. (**D**) Colony formation assay showing the proliferation of cells transfected with *FDPS* siRNA. The diameter of each well is 35 mm. Lower panels show ×2 magnified views of the boxed regions in the upper panels. Scale bar = 3.5 mm. In all bar graphs, individual data points (*n* = 3) are shown as dots. Statistical significance: * *p* < 0.05; ** *p* < 0.01; *** *p* < 0.001.

To investigate the functional role of FDPS in endometrial cancer, FDPS-specific siRNA was transfected into Ishikawa cells to suppress FDPS expression. The Ishikawa cells were selected for this study because they represent a well-differentiated, grade 1 endometrioid adenocarcinoma cell line, reflecting early-stage type 1 endometrial cancer. Compared with cells transfected with the negative control (NC) strand, FDPS-siRNA-transfected cells exhibited a significant reduction in FDPS mRNA and protein levels (Figure 6A,B).

Cell viability analysis using the CCK-8 assay revealed that FDPS-knockdown cells showed consistently lower absorbance values compared with both NC and mock groups (Figure 6C). Furthermore, in the colony formation assay, FDPS-knockdown cells formed significantly fewer colonies than NC and mock groups (Figure 6D). These findings indicate that FDPS plays a crucial role in both short-term cell viability and long-term proliferation of Ishikawa cells, suggesting its importance in maintaining the proliferative capacity of endometrial cancer cells.

## 3. Discussion

In this study, we aimed to elucidate the expression pattern, regulatory mechanism, and functional role of FDPS in the uterus and endometrial cancer. We found that FDPS expression in the mouse uterus was upregulated in response to estrogen signaling. Notably, FDPS expression increased significantly during the proestrus stage, when estrogen levels are highest, and in ovariectomized (OVX) mice 24 h after estrogen administration. This timing is consistent with estrogen-dependent transcriptional activation, as meaningful changes in gene expression generally become evident at approximately 24 h following estrogen stimulation, reflecting the time point at which transcriptional regulation is fully manifested [3]. This induction was mediated through estrogen receptor alpha (ERα).

FDPS is a key regulatory enzyme in the mevalonate pathway, catalyzing the synthesis of farnesyl pyrophosphate (FPP), a precursor of cholesterol and various lipid metabolites [5]. Cholesterol is an essential precursor for estrogen biosynthesis, and intracellular cholesterol levels are a critical determinant of estrogen synthesis efficiency [10]. Conversely, estrogen has been reported to modulate cholesterol metabolism by regulating the expression of enzymes involved in cholesterol synthesis and transport [11]. Previous studies have also demonstrated reciprocal interactions between intracellular cholesterol accumulation and enhanced estrogen biosynthesis [12]. Therefore, the estrogen-dependent regulation of FDPS observed in this study suggests a potential feedback relationship between mevalonate pathway-mediated cholesterol metabolism and estrogen signaling.

In our study, FDPS expression was predominantly observed in luminal and glandular epithelial cells (LE and GE) of the endometrium. As a central enzyme in the mevalonate pathway, FDPS is generally expressed at higher levels in metabolically active tissues such as liver, brain and bone [13,14]. Consistently, previous studies have reported high expression of *Fdps* and other sterol biosynthesis-related genes in the luminal epithelium at day 3.5 of pseudopregnancy, which corresponds to the implantation window in mice [15]. These findings support the epithelial-specific expression pattern of FDPS observed in our study. Furthermore, FDPS expression has also been reported in the epithelial cells of other organs [16], suggesting that FDPS may contribute to epithelial metabolic regulation. In particular, our findings that FDPS expression peaks at 24 h following estrogen treatment and is suppressed by ICI 182,780 are consistent with previous reports demonstrating that late transcriptional responses in the uterus are predominantly regulated by epithelial ERα through genomic estrogen signaling [3]. However, the precise molecular mechanisms underlying the epithelial regulation of FDPS by estrogen signaling remain to be further elucidated.

Aberrant expression of FDPS has been associated with various diseases, particularly cancers [17,18]. Several human malignancies exhibit elevated FDPS expression. Dysregulated FDPS expression has also been associated with a poor prognosis for patients, suggesting that FDPS may serve as a potential biomarker for tumor progression and outcome. For instance, elevated FDPS expressions at both the mRNA and protein levels have been observed in breast cancer, a representative estrogen-responsive malignancy [19]. However, research into FDPS expression and its role in endometrial cancer (EC) is limited. EC originates from endometrial epithelial cells [8], and in our study, FDPS was primarily expressed in these components. This finding suggests that FDPS expression reflects the cellular composition of EC and altered metabolic activity in epithelial cells. Given that previous reports have shown an association between elevated FDPS expression and tumor progression and poor prognosis in other malignancies [20,21], FDPS expression in EC may also contribute to its pathological transformation and progression.

To further characterize FDPS expression in normal and malignant human endometrium, we performed an immunofluorescence analysis using human endometrial tissue microarrays (TMAs). In normal endometrium, FDPS expression was high in glandular epithelial cells during the proliferative phase but was markedly reduced in the secretory phase. This pattern was consistent with our findings in the mouse uterus, suggesting that FDPS expression in the human endometrium may also be influenced by estrogen signaling. However, additional studies employing hormonally controlled models are required to confirm whether FDPS is directly regulated by estrogen in the human endometrium.

Based on these findings, we examined the expression of FDPS in type 1 endometrioid adenocarcinoma, which is characterized by estrogen dependency. FDPS expression was significantly elevated in well-differentiated grade 1 endometrioid adenocarcinoma, particularly in epithelial cells. However, it gradually decreased with increasing tumor grade. Grade 1 and 2 tumors are well-differentiated, characterized by the maintenance of glandular structures (accounting for 50–95% of the tissue), whereas grade 3 tumors are poorly differentiated, with a predominance of solid growth patterns and a loss of glandular features [7]. Type 1 ECs are generally estrogen-dependent, a characteristic typically maintained in grade 1–2 tumors [8]. Therefore, the observed decrease in FDPS expression in grade 3 tumors likely reflects both the loss of estrogen dependency and the reduction of epithelial-derived glandular structures during tumor progression. While FDPS is often reported to increase with higher malignancy and poor prognosis in other cancers [20,21], our results show a unique pattern in EC. This discrepancy may stem from the tissue-specific metabolic requirements of the endometrium, where FDPS-mediated pathways are specifically harnessed for estrogen-driven physiological and pathological proliferation. These results suggest that FDPS functions as a context-dependent mediator within the estrogen-responsive environment of the endometrium.

To investigate the functional role of FDPS in EC, we utilized Ishikawa cells, a grade 1, estrogen-responsive EC cell line [7]. Based on the high FDPS expression observed in TMAs, we performed siRNA-mediated FDPS knockdown and evaluated the resulting cellular changes. Suppression of FDPS expression significantly reduced cell proliferation in both short-term (within 72 h) and long-term (approximately 10 days) assays. Taken together with previous studies showing that FDPS overexpression promotes cell proliferation in other cancer cell lines [14,18], our knockdown data further support the functional role of FDPS in regulating cell proliferation in EC.

Overall, our findings show that FDPS is a factor related to metabolism that is regulated by estrogen signaling and is highly expressed in well-differentiated type 1 endometrial carcinomas. FDPS and estrogen may exert mutual regulatory interactions that influence endometrial proliferation and tumor growth, highlighting FDPS as a potential therapeutic target in estrogen-dependent EC. FDPS may also have a potential role in other estrogen-driven proliferative disorders. For example, endometrial atypical hyperplasia is well recognized as a precursor lesion of type 1 EC and is characterized by excessive estrogen-induced epithelial proliferation [22]. In light of our findings that FDPS is regulated in an estrogen-dependent manner in EC, dysregulation of FDPS may contribute to the early stages of neoplastic transformation. Accordingly, FDPS may function not only in the progression of endometrial cancer but also as a common molecular regulator in estrogen-dependent gynecological disorders.

In summary, our findings suggest that FDPS functions as a metabolic regulator whose expression is controlled by estrogen-ERα signaling in the normal uterus and contributes to cell proliferation and tumor progression in pathological contexts. Future studies should elucidate the specific signaling pathways through which FDPS regulates EC cell proliferation. A deeper understanding of the function of FDPS will provide critical insights into the interplay between hormones and metabolism in endometrial cancer and aid in the development of new therapeutic strategies.

## 4. Materials and Methods

### 4.1. Bioinformatic Analysis

Public datasets were reanalyzed for bioinformatic analysis. Bulk RNA-seq data of mouse uteri across the estrous cycle (GSE241420), microarray datasets of mouse uteri treated with estradiol (E_2_) for 2 and 24 h (GSE53812), and uteri from the ERα knockout (ERαKO) mouse (GSE23072) were downloaded and analyzed using the publicly available web tool GEO2R (https://www.ncbi.nlm.nih.gov/geo/info/geo2r.html, accessed on 5 July 2025). Genes showing significant differential expression were selected based on FDR < 0.05 and |logFC| > 1.0. The files used to generate the heatmap are provided in the Supplementary Files S1–S6.

### 4.2. Animals

All animal experiments were approved by the Institutional Animal Care and Use Committee (IACUC, Approval No. KU24078, approved on 14 June 2024) and were conducted in accordance with institutional guidelines for the Care and Use of Laboratory Animals. Female CD-1 mice (5 weeks old) were purchased from JA BIO (Suwon, Republic of Korea). The animals were maintained under standardized environmental conditions (12-h light/dark cycle, constant temperature) and had free access to food and water. The housing and experimental conditions were consistent with those previously described [23].

### 4.3. Hormone Treatments

After a two-week acclimation period, ovariectomy was performed on fifteen female CD-1 mice to eliminate endogenous hormonal activity. Mice were anesthetized, and a small incision (~1 cm) was made in the flank to surgically remove both ovaries and oviducts. Following a 14-day recovery period, the mice were randomly divided into five groups (*n* = 3 per group). Each group received an intraperitoneal injection of β-estradiol (E_2_; E8875, 300 ng/mouse, Sigma-Aldrich, St. Louis, MO, USA) at a dose of 300 ng per mouse. Mice were euthanized by cervical dislocation at predetermined time points (0, 6, 12, 24 and 48 h after injection), and uterine tissues were collected. The uteri were rinsed in cold DPBS, and one uterine horn from each mouse was used for protein analysis, while the other was bisected—one half for RNA analysis and the other for fixation for subsequent immunostaining.

To further assess the involvement of nuclear estrogen receptors, twelve ovariectomized CD-1 mice were randomly divided into four groups (*n* = 3 per group): a vehicle control group, a group treated with ICI 182,780 (Fulvestrant; HY-13636, MedChem Express, Shanghai, China), an ER antagonist, a group treated with E_2_ alone, and a group treated with both ICI and E_2_. The group treated with ICI received a subcutaneous injection of ICI (0.5 mg/mouse) 30 min prior to E_2_ administration. 24 h after E_2_ treatment, the mice were euthanized by cervical dislocation, and uterine tissues were collected for subsequent analyses.

To evaluate the potential effect of progesterone, an additional group of ovariectomized CD-1 mice received a single subcutaneous injection of progesterone (P0130, 2 mg/mouse, Sigma-Aldrich, St. Louis, MO, USA). Uterine tissues were collected 24 h after treatment.

### 4.4. RNA Preparation, RT-PCR and qRT-PCR

RNA extraction procedures were performed with reference to the method described by Kim et al., with minor modifications as detailed below [24]. For all experiments, uterine tissues were collected from three independent mice per experimental group (*n* = 3). Total RNA was isolated from the collected uterine tissues using the RNeasy Total RNA Isolation Kit (74106, Qiagen, Hilden, Germany). The extracted RNA (1 μg) was reversed-transcribed into cDNA using the SensiFAST™ cDNA Synthesis Kit (BIO-65054, Bioline, London, UK). The synthesized cDNA was subsequently used for both RT-PCR and qRT-PCR analyses.

RT-PCR was carried out with Solg™ Taq DNA Polymerase (SolGent, Daejeon, Republic of Korea) using a ProFlex PCR system (Thermo Fisher Scientific, Waltham, MA, USA). PCR products were visualized by 2% agarose gel electrophoresis after staining with Dyne LoadingSTAR (A750, Dyne Bio, Seoul, Republic of Korea). Ribosomal protein L7 (*Rpl7*) and actin beta (*ACTB*) served as endogenous control genes. Gels were imaged and analyzed using the ChemiDoc™ XRS+ System (Bio-Rad, Hercules, CA, USA).

qRT-PCR was carried out with each biological sample analyzed in technical replicates using the PowerUp™ SYBR Green Master Mix (A25780, Thermo Fisher Scientific) on a QuantStudio™ 1 Real-Time PCR Instrument (Applied Biosystems, Foster City, CA, USA), following the manufacturer’s protocol. Relative mRNA expression levels were calculated using the ΔΔCT and 2^−ΔΔCT^ methods, normalized to *Rpl7* or *ACTB* expression. The primer list used in this study and RT-PCR conditions are provided in Supplementary Figure S3.

### 4.5. Western Blot Analysis

For all experiments, uterine tissues were collected from three independent mice per experimental group (*n* = 3). Proteins were extracted from uterine tissues using RIPA buffer (R0278, Sigma-Aldrich) supplemented with a protease inhibitor cocktail (HY-K0010, MedChem Express). A total of 20 μg of extracted protein was separated by 12% SDS-PAGE under a constant current of 60 mA for 1 h. The separated proteins were subsequently transferred to a PVDF membrane (Bio-Rad, Hercules, CA, USA) at 350 mA for 1 h. The membrane was blocked with 5% skim milk (232100, BD Difco, Sparks, MD, USA) at room temperature for 2 h and subsequently incubated with primary antibodies: anti-FDPS (1:1000, ab153805, Abcam, Cambridge, UK) and anti-β-actin (1:10,000, sc-47778 HRP, Santa Cruz, Dallas, TX, USA). Protein detection was performed using HRP-conjugated secondary antibody (1:5000, G21234, Invitrogen, Waltham, MA, USA), and signals were revealed using the SuperSignal™ West Pico PLUS Chemiluminescent Substrate (Thermo Fisher Scientific). The resulting chemiluminescent signals were captured using the ChemiDOC™ XRS+ system (Bio-Rad), and FDPS expression levels were quantified using ImageJ (Fiji) software (version 2.11.0), normalized to β-actin.

### 4.6. Immunofluorescence Staining

Immunofluorescence staining procedures were performed with reference to the method described by Hwang et al. [25], with minor modifications as detailed below. The collected uterine tissues were washed with cold DPBS and fixed in 4% paraformaldehyde (PFA) at 4 °C overnight. The fixed tissues were processed for paraffin embedding and sliced into 5-μm-thick sections. After deparaffinization and rehydration, heat-induced antigen retrieval was carried out in 10 mM sodium citrate buffer (pH 6.0) at 95 °C for 20 min. Sections were blocked at room temperature for 2 h in a blocking buffer containing 0.1% Triton X-100 and 5% donkey serum (ab7475, Abcam). Slides were incubated with the primary antibody anti-FDPS (1:200, ab153805, Abcam) at 4 °C for 16 h, washed with 1X PBS, and then incubated with Alexa Fluor 647-conjugated secondary antibody (1:500, A-31573, Thermo Fisher Scientific) for 1 h at room temperature. Slides were mounted using DAPI-containing mounting medium (ab104139, Abcam) and imaged using a confocal laser scanning microscope (ZEISS, Oberkochen, Germany). Images were acquired using ZEN 2012 software (Carl Zeiss Co. Ltd., Oberkochen, Germany) at 20× and 40× magnifications. Three-slice z-stacks were captured and merged using maximum intensity projection. Relative fluorescence intensity was quantified using ImageJ (Fiji).

Human endometrial tissue microarrays (TMAs) embedded in FFPE blocks were obtained from US Biomax, Inc. (Rockville, MD, USA). Immunofluorescence staining was performed as described above. The catalog numbers of the TMAs used were as follows: normal tissue, UTN801; endometrial cancers, BC09012b.

### 4.7. Ishikawa Cell Culture and siRNA Transfection

Ishikawa cells were maintained in phenol-red-free DMEM (Gibco, Thermo Fisher Scientific) containing 10% fetal bovine serum (FBS; Gibco, Thermo Fisher Scientific) and 1% penicillin-streptomycin (P/S; HyClone, Cytiva, Malborough, MA, USA) under humidified conditions (37 °C, 5% CO_2_).

siRNAs were synthesized by Bionics (Seoul, Republic of Korea), and the sequences used for FDPS and negative control (NC) siRNAs were as follows:

*FDPS*-sense: 5′-GUUCCUAUCAGACUGAGAUTT-3′;

*FDPS*-antisense: 5′-AUCUCAGUCUGAUAGGAACTT-3′;

NC-sense: 5′-UUCUCCGAACGUGUCACGUTT-3′;

NC-antisense: 5′-ACGUGACACGUUCGGAGAATT-3′.

Transfection was performed using Lipofectamine™ RNAiMAX (13778150, Invitrogen, Thermo Fisher Scientific) in accordance with the manufacturer’s protocol. Ishikawa cells were cultured in 6-well plates until approximately 70% confluency, then transfected with FDPS or NC siRNAs at a final amount of 25 pmol per well, following the standard protocol. Cells were harvested 48 h post-transfection for subsequent analyses.

### 4.8. CCK-8 Assay

After transfection, Ishikawa cells were cultured for 24 h and seeded into 96-well plates at 5000 cells/well. At 24, 48, and 72 h post-transfection, cell viability was evaluated with the cell counting kit 8 (CCK-8; 96992, Sigma-Aldrich), modified from a previously described WST-based colorimetric assay [26]. After adding CCK-8 reagent (10 μL per well), the cells were incubated at 37 °C for 2 h. Absorbance at 450 nm was read using a microplate reader (VersaMax, Molecular Devices, San Jose, CA, USA).

### 4.9. Colony Formation Assay

After transfection, Ishikawa cells were cultured for 24 h and seeded into 6-well plates at 1000 cells/well. The culture medium was replaced every 3 days, and cells were maintained for 10 days. After incubation, the cells were rinsed twice with DPBS and subsequently fixed with 4% PFA for 15 min, stained with 0.1% crystal violet (DAEJUNG, Siheung, Republic of Korea) for 5 min, and washed again with DPBS. After air-drying the wells thoroughly, colony images were captured, and colony numbers were quantified using ImageJ (Fiji).

### 4.10. Statistical Analysis

Data were obtained from three independent biological replicates (*n* = 3) under identical experimental conditions. Individual data points represent values from each replicate. All data are presented as mean ± SEM (standard error of the mean). Statistical significance was evaluated by one-way ANOVA, followed by Tukey’s post hoc test for multiple comparisons, with *p* < 0.05 regarded as significant.

## Data Availability

The data supporting the findings of this study are available from the corresponding author upon reasonable request.

## Supplementary Materials

The following supporting information can be downloaded at: https://www.mdpi.com/article/10.3390/ijms27031559/s1.

## Abbreviations

The following abbreviations are used in this manuscript:

CE	Cornified epithelial cell
E_2_	β-estradiol
EC	Endometrial cancer
ER	Estrogen receptor
FDR	False discovery rate
GE	Glandular epithelial cell
LE	Luminal epithelial cell
LK	Leukocyte
NE	Nucleated epithelial cell
OVX	Ovariectomized
