# Supplementary material for: Estrogen-Dependent Regulation of FDPS in the Mouse Uterus and Its Expression in Endometrial Cancer
Source: Int J Mol Sci. 2026 Feb 5;27(3):1559. doi: 10.3390/ijms27031559 (PMC12898277; doi:10.3390/ijms27031559)
Supplement: Supplementary file 1 [file ijms-27-01559-s001.zip › Supplementary Figure and Files/Supplementary Figure_revision.pdf]

Supplementary Figure S1. Quantification of FDPS immunofluorescence intensity in the mouse uterus.

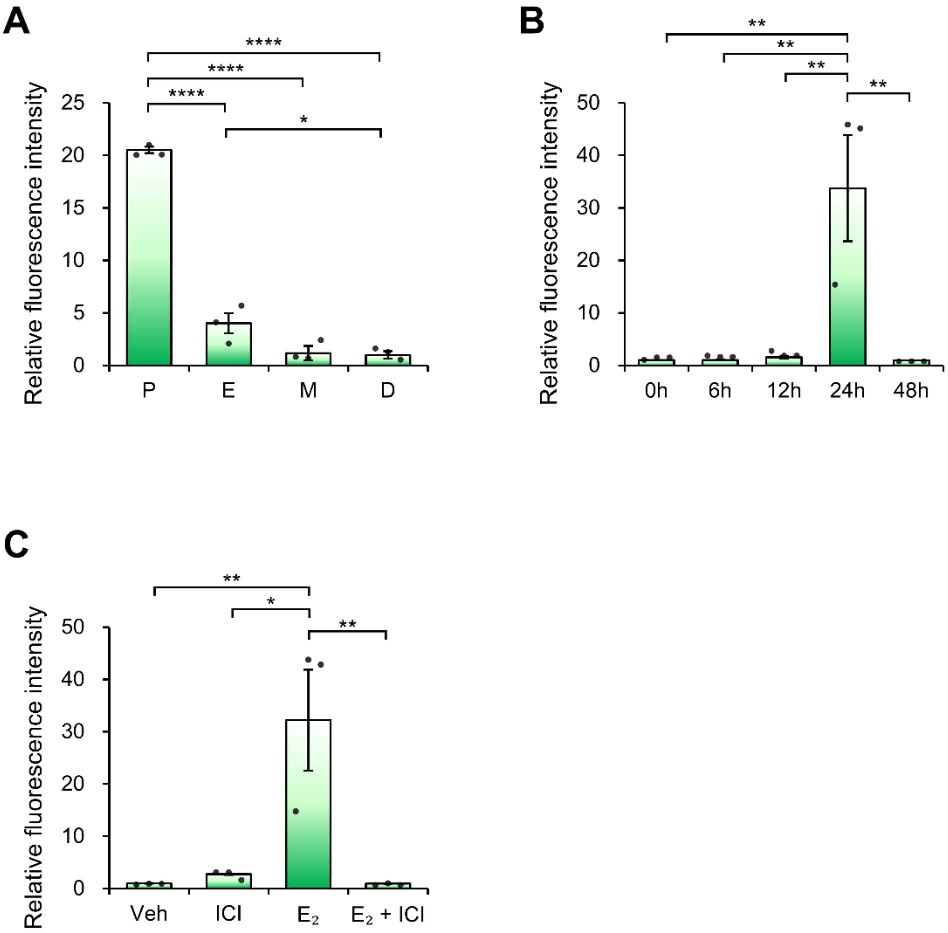

(A) Relative fluorescence intensity of FDPS across the estrous cycle. (B) Relative fluorescence intensity of FDPS after E_2 treatment. (C) Relative fluorescence intensity of FDPS 24 h after treatment with vehicle, ICI, E_2 and E_2+ICI. Individual data points (n = 3) are shown as dots. Statistical significance: *p < 0.05; **p < 0.01; ****p < 0.0001.

Supplementary Figure S2. *Fdps* expression 24 hours after E_2 and P_4 treatment.

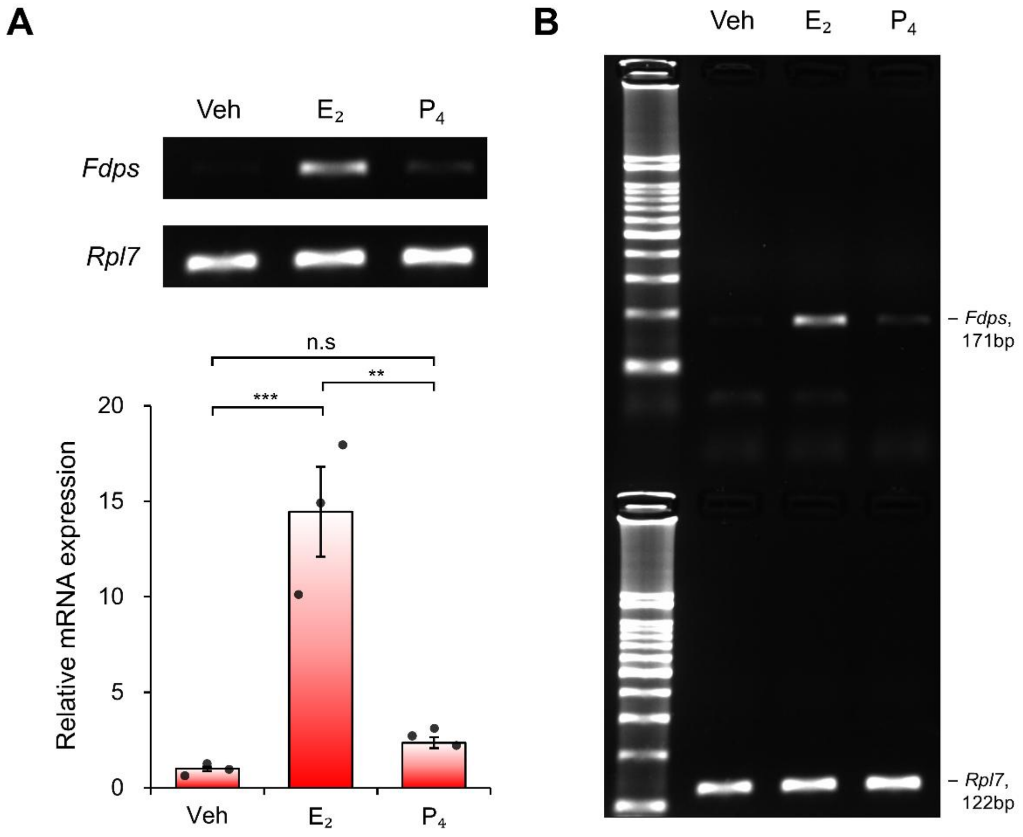

(A) RT-PCR and qPCR analyses of *Fdps* in OVX mouse uteri after E_2 and P_4 treatment for 24 hours. *Rpl7* was used as an internal control. Veh, vehicle; E_2, estrogen; P_4, progesterone. Individual data points (n = 3) are shown as dots. Statistical significance: n.s, not significant; **$p < 0.01$; ***$p < 0.001$. (B) Full-length agarose gel image of RT-PCR products showing all lanes and molecular weight markers.

Supplementary Figure S3. Primer list and RT-PCR conditions.

Gene	Sequence (5'-3')	Product Size (bp)	Cycles	Annealing Temperature
Fdps	Forward: GTATCAGAAGCCAGGCATAGG<br>Reverse: AGGTCTAGAGTCTGCCCGAT	171	30	55℃
Ltf	Forward: GGGACAGGAGCCAACAAATG<br>Reverse: ACTGGTCCCTTTCGGCTTTA	157	30	60℃
Rpl7	Forward: GTCTTCCCTGTTGCCAGCAT<br>Reverse: GGAAACGCTTCAAGGAAGCAA	122	26	60℃
FDPS	Forward: GTGGTAGTAGCATTCCGGGA<br>Reverse: TTCAGCAGGCGGTAGATACA	242	30	55℃
ACTB	Forward: GAGAAAATCTGGCACCACACC<br>Reverse: GATAGCACAGCCTGGATAGCA	176	26	60℃

The RefSeq accession numbers for each gene are as follows: *Fdps* (*Mus musculus*), NM_001253751.1; *Ltf* (*Mus musculus*), NM_008522.3; *Rpl7* (*Mus musculus*), NM_011291.5; *FDPS* (*Homo sapiens*), NM_002004.4; *ACTB* (*Homo sapiens*), NM_001101.5.
